# Screening for Cd-Safe Cultivars of Chinese Cabbage and a Preliminary Study on the Mechanisms of Cd Accumulation

**DOI:** 10.3390/ijerph14040395

**Published:** 2017-04-07

**Authors:** Jingjie Wang, Nan Yu, Guangmao Mu, Kamran I. Shinwari, Zhenguo Shen, Luqing Zheng

**Affiliations:** College of Life Sciences, Nanjing Agricultural University, Nanjing 210095, Jiangsu, China; 2016816157@njau.edu.cn (J.W.); 2014816158@njau.edu.cn (N.Y.); 2014116044@njau.edu.cn (G.M.); kamishin@yahoo.com (K.I.S.); zgshen@njau.edu.cn (Z.S.)

**Keywords:** Chinese cabbage, Cd, cultivars screening, low-accumulation, gene expression

## Abstract

With the rapid progress of industrialization, the effects of environmental contamination on plant toxicity, and subsequently on human health, is a growing concern. For example, the heavy metal pollution of soil such as that caused by cadmium (Cd) is a serious threat. Therefore, screening for pollution-safe edible plants is an essential approach for growing plants under heavy metal-contaminated soils. In the current study, 35 Chinese cabbage (*Brassica pekinensis* L.) cultivars were selected with the aim of screening for Cd-safe cultivars (CSCs), analyzing their safety, and exploring the mechanism of Cd accumulation. Our field-culture experiments revealed that the Cd content in the edible parts of the cultivars were varied and were determined to possibly be CSCs. Hydroponics experiments were used to simulate six different degrees of soil contamination (high and low Cd concentrations) on possible CSCs. The results indicated a significant difference (*p* < 0.05) in Cd concentration in the cultivars, and verified the safety of these possible CSCs. The analyses of the transport coefficient and expression levels showed that the differences in Cd accumulation among the Chinese cabbage cultivars were related to the expression of genes involved in absorption and transport rather than a root-to-shoot translocation limitation.

## 1. Introduction

The intensification of soil pollution with heavy metals (HMs) has become a worldwide concern, instigated by numerous anthropogenic activities such as the large spreading of fertilizers, use of pesticides, burning of fossil fuels, mining and smelting, treatment of municipal wastes, and the creation of sewage sludge [1]. In China, reports show that more than 200,000 ha of farmland soil have been contaminated by cadmium (Cd). Furthermore, the 1.46 million tons of agricultural products harvested from these soils surpass the national standards for food safety of China (NSFSC) [2]. The Environmental Quality Standards for Soil of China (EQSSC) states that the maximum permissible concentration (MPC) of total Cd (GB15618-2008) in vegetable soils is 0.3 mg/kg (pH < 5.5–6.5). Furthermore, with regard to the maximum level of contaminant in food (GB2762-2012) allowed by the NSFSC, the MPC standard of Cd in vegetables for safe consumption is 0.2 mg/kg fresh weight [2]. Cd accumulates in the edible parts of plants, eventually causing acute health problems in humans via the food chain [3]. The persistent use of unsafe vegetables leads to the gradual accumulation of HMs, and with respect to toxic Cd, leads to excessive accretion in the human body that results in damage to glomerular and renal tubules, a decrease in bone mineral density, osteoporosis, and an increased risk of cancer [4,5,6].

Previous studies have shown that the ability of HMs to accumulate in different crops and vegetables is variable. It has been established that leafy vegetables store a higher amount of HMs, followed by root vegetables and legumes [7]. The accretion of HMs in plants differs prominently not only among plant species but also among cultivars of the same species. For example, Arao et al. [8] planted 49 rice cultivars in Cd-contaminated soil and found that the variation in Cd content was more remarkable among cultivars than the differences between the stem and roots of the same cultivar. Substantial differences in Cd accumulation within the tubers of 14 common potato cultivars were reported by McLaughlin et al. [9]. In addition, the concentration of Cd among cultivars of other crops has also been determined. In recent years, the screening of different crop cultivars as a measure for food safety has been explored. This could be a substitute for lowering the risk of soil Cd entering the human food chain, without the need for ploughing the land. Specifically, Cd-safe cultivars (CSCs), which accrue Cd at low amounts for innocuous consumption even when grown in polluted environment or soils, could be used [10].

HMs in the soil are absorbed by the roots of plants, after which they are transferred to their shoots, processes that are facilitated by HM transporters. To date, transporters with specificity and selectivity for Cd have not been found in plants [11,12,13,14]. Higher plants absorb Cd predominantly via the transporters of cationic metals such as iron (Fe), manganese (Mn), and zinc (Zn) [15,16]. To date, there have been many gene families involved in plant Cd uptake and transport, such as ATP-binding cassette transporters (ABC), natural resistance-associated macrophage protein (*Nramp*), heavy metal ATPases (HMA), and metal transporter proteins (MTPs). The *Nramp* family encodes Mn and Fe transporters that are expressed on cell membranes. In rice, Cd accretion was considerably reduced in *Nramp5* knockouts [17]. In Arabidopsis, *Nramp3* overexpression increased Cd sensitivity, signifying the involvement of *Nramp3* in Cd transport in Arabidopsis [18]. Moreover, under Cd toxicity the expression level of correlated Cd responsive genes can be affected [19]. For example, the nitrate transporter 1 (*NRT1*) is a gene family that encodes proteins for nitrate transport and nitrate reallocation under stress, and *NRT1.8* is the nitrate upregulated gene expressing in the xylem of the plant roots [20,21]. In Arabidopsis, it has been shown that upregulation of *NRT1.8* to certain extant can decrease the absorption of Cd in root and slow down the shoot growth [21]. However, little is known about the differences in expression of Cd-induced genes in different Chinese cabbage cultivars, and the mechanisms by which these plants absorb and translocate Cd at the molecular level.

Chinese cabbage (*Brassica pekinensis* L.) is a chief cruciferous vegetable that regularly grows in China and other East Asian countries. Chinese cabbage is rich in vitamins, possesses a variety of nutrients, and has been an essential oil crop in regions of southern China due to its oil-rich seeds. However, the Cd content in Chinese cabbage, particularly in those plants grown in contaminated soil, is a latent threat to food safety and human health. Therefore, there is a pressing need to develop reasonable farming approaches to produce vegetables with low levels of HMs in polluted regions [22]. The Qixia District in Nanjing City (Jiangsu Province, China) is an important and modern industrial area where Zn and lead (Pb) mining and smelting activities produce wastes instigating the release and accrual of noxious Cd content, which causes HM contamination in suburb soils, and consequently, a substantial increase in toxic metals in vegetables such as Chinese cabbage and other crops cultured nearby. Hence, the consumption of these foods is considered unsafe for the large population of consumers [23,24,25].

The goal of this study was to reduce the hazards of Cd to the wellbeing of humans (as well as animals). To this end, we identified CSCs from 35 Chinese cabbage cultivars by performing field-culture experiments in our study area, and then verified the low-Cd accumulation potential of CSCs by conducting a hydroponic experiment. To understand the differences in Cd uptake among the different cultivars, we analyzed the Cd translocation factors (TFs) or transport coefficients and expression levels of the metal transporter genes involved in Cd uptake. Our work makes a significant scientific contribution to research by not only revealing the mechanism of low-Cd accumulation in CSCs, but also by demonstrating how to screen for CSCs via molecular methods.

## 2. Materials and Methods

### 2.1. Field Site and Soil Analysis

The experiment was carried out at contaminated land of peasant plots in the suburb of Qixia District (32°09′10.18″ N, 118°57′15.07″ E), located in the north of Nanjing City, Jiangsu Province, China. The contents of available heavy metals in soils were determined by ICP-OES (PerkinElmer, Waltham, MA, USA) and ICP-MS (PerkinElmer, Waltham, MA, USA), with DTPA (Diethylenetriaminepentaacetic acid) as extractant, and the details refer to Li et al. [26]. The methods of measuring soil pH refer to ISO 2005:10390 and the organic matter, available phosphorus (P), available potassium (K), and available ammonium were measured with a soil nutrient analyzer (TPY-16A) (Tuopu, Hangzhou, China) [27].

### 2.2. Field-Culture Experiment

The seeds of 35 Chinese cabbage cultivars (Table 1) were sowed directly into the soil (with sowing time period from mid-April to late May 2015, mean temperature 22–25 °C) in an area that had been divided into a 40 × 40 cm^2^ area (for each cultivar). Forty days after sowing, the edible parts (shoots) of plant were harvested for Cd content analysis. The field-culture experiment of Chinese cabbage was repeated three times to screen out varieties with stable high-Cd and low-Cd accumulation potentials.

### 2.3. Hydroponics Experiment

According to the available Cd concentration in soil (1.73 mg/kg, about 15 μM) and the MPC of total Cd (0.3 mg/kg, about 2.6 μM) in EQSSC (GB 15618-2008), six treatments, including a control (C, without Cd addition) and T_1–5_ (Cd concentrations: 10, 20, 0.02, 0.2, and 2 μM, respectively) were arranged and applied in a hydroponics experiment. T_1_ and T_2_ stand for high-Cd contamination treatments, while T_3–5_ stand for low Cd contamination treatments. For selection of cultivars, i.e., subsequently confirming from the field-culture experiments (after three repetitions), only the cultivars Hualvqingxiu (HLQX) and Canbai (CB) selected had stable and low Cd accumulation in comparison to the other tested cultivars, while Dongfang2hao (DF2H) had persistent and high Cd accumulation. So seeds of HLQX, CB as low Cd cultivars, and DF2H as high Cd cultivars were selected from the field-culture experiment and were sown in a seed tray matrix (as replicates) containing 1/3 vermiculite, 1/3 nutrient soil, and 1/3 perlite (placed at 25 °C and in 16/8 h day/night conditions in greenhouse). After 14 days, the seedlings with good and consistent growth were transplanted into Hoagland’s solution (5 L). Four days after transplanting, Cd (CdCl_2_) was added into the culture solution according to the above-mentioned (T_1–5_) treatments for 14 days, after that the different cabbage plants treatments were taken for subsequent analysis. 

### 2.4. Cd Content Analysis

The shoots and roots of the harvested cabbage cultivar samples were rinsed with 5 mM CaCl_2_ solution, and then carefully washed with deionized water [28]. The samples were then dried in an oven at 80 °C until dried completely. The dried samples were ground to a powder, and 0.2 g were weighed and transferred to digestion tubes for digestion with a 5 mL solution containing 87% concentrated HNO_3_ and 13% concentrated HClO_4_ (v/v). After complete digestion, 10 mL of 2.5% HNO_3_ in the samples were added and mixed well, then dissolved in a water bath at 60 °C for 30 min. Finally, the Cd content of the samples was determined by ICP-MS (PerkinElmer, Waltham, MA, USA) [29] and the Cd translocation factors (TFs, defined as the ratio of the concentration of Cd in plant shoots to the concentration of Cd in plant roots) was analyzed [22]. 

### 2.5. RNA Isolation and Quantitative PCR for Cd-Uptake-Related Genes

To check the expression of Cd-uptake-related genes (*Nramp3* and *NRT1.8*), roots of DF2H, CB, and HLQX collected from 20-day-old plants were used to investigate mRNA expression throughout the study. The selected cultivars DF2H, CB, and HLQX were hydroponically cultivated, and the culture conditions were the same as mentioned above. Four days after transplanting, the plants were cultured for 24 h under a hydroponic solution of the control condition. The fresh roots were separated after washing and were then air-dried. The samples were wrapped with aluminum foil after being ground in liquid nitrogen.

Total RNA was extracted by using MiniBEST Plant RNA Extraction Kit (TaKaRa, Tokyo, Japan), and the first-strand cDNAs were synthesized from DNaseI-treated total RNA using PrimeScript™ RT reagent Kit (TaKaRa, Tokyo, Japan) with gDNA Eraser (TaKaRa, Tokyo, Japan) according to the manufacturer’s instructions. Real-time PCR was performed with SYBR^®^ Premix Ex Taq II (Tli RNaseH Plusplus) (TaKaRa, Tokyo, Japan) using a 7500 PCR system (Applied Biosystems, Foster City, CA, USA). PCR amplification was performed in a total volume of 20 μL including 10 μL SYBR^®^ Premix Ex Taq II (Tli RNaseH Plus), 30 ng of cDNA, and 0.4 μM gene-specific primers i.e., *Nramp3* (CabbageG_a_f_g014420) forward, 5′-GATCCAGGAAACCTCGAAGG-3′; reverse 5′-AGAGCTGAACAAGAAGCCCC-3′ and *NRT1.8* (CabbageG_a_f_g017074) forward, 5′-GATGGGACAAGACAATGCAGAAG-3’; reverse 5′-GAAAGCACCAAGCAAAGAGAAGA-3′. The *Actin* gene was amplified by primers forward, 5′-GTTGCTATCCAGGCTGTTCT-3′; and reverse, 5′-AGCGTGAGGAAGAGCATAAC-3′ and used as an internal positive control for quantification. The PCR condition were optimized as preliminary denaturation at 95 °C for 30 s, followed by 95 °C for 5 s, and 60 °C for 34 s (in a 40-cycle reaction), then followed by dissociation stage. Quantification of gene expression was performed using the comparative 2^−ΔΔCt^ method, and three independent biological replicates were performed for each sample [30].

### 2.6. Statistical Analysis

All data were analyzed with software SPSS 19.0 (IBM, Armonk, NY, USA), Origin 8.0 (OriginLab, Hampton, MA, USA), and Excel 2010 (Microsoft, Redmond, WA, USA) for Windows 7. Differences in gene expression among cultivars were analyzed by a one-way ANOVA (*p* < 0.05) followed by a Tukey’s post-hoc test. Correlation analysis (using Pearson product–moment correlation) was conducted using SPSS 19.0.

## 3. Results

### 3.1. Soil Characterization

The content of the soil was a loam (pH 6.12) with organic matter, ammonium, available P, and available K contents of 5.12, 25.67, 37.54, and 49.89 mg/kg DW, respectively (Table 2). The soil was extremely contaminated with superfluous Cd; concentrations of total Cd and available Cd were 2.42 and 1.73 mg/kg DW, respectively (Table 2). According to the EQSSC (GB 15618-2008), the tested soil was considered a heavy Cd-contaminated acreage.

### 3.2. Cd Accumulation in the Field-Culture Experiment

**Figure 1:**
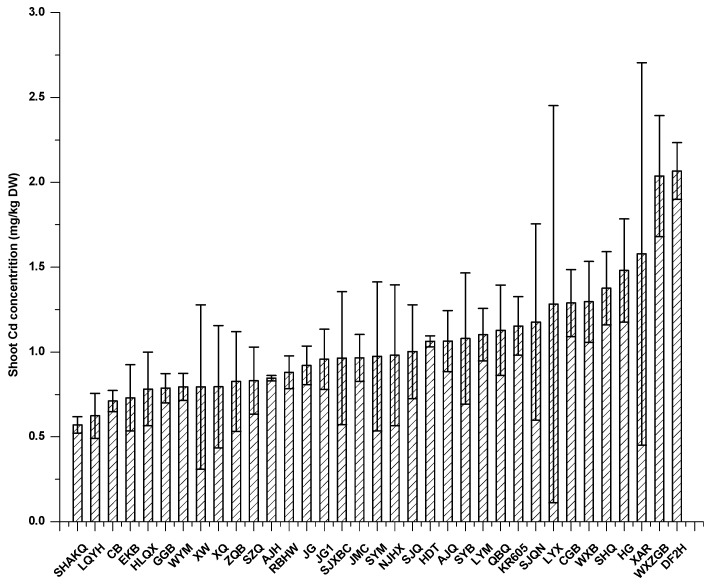
Cd content in the shoots of 35 varieties of non-heading Chinese cabbage.

To investigate whether Cd-contaminated soil increased Cd accumulation in vegetables and threatened human health, 35 cultivars of Chinese cabbage were planted in this HM accumulation study. The Cd concentrations in vegetable shoots ranged from 1.05 to 3.51 mg/kg DW (Figure 1). Cd accumulation varied significantly in the different cultivars of cabbage. From three repeated experiments in field, we screened two low-Cd-accumulation cultivars Hualvqingxiu (HLQX) and Canbai (CB) and a high-Cd-accumulation cultivar Domngfang2hao (DF2H), which was selected as a control for analysis of mechanism and toxicity. 

### 3.3. Differences in Cd Uptake of Three Cultivars under High Cd Concentration

**Figure 2:**
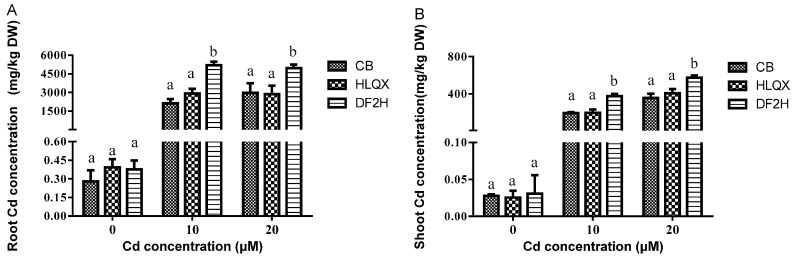
Cd content in the (**A**) roots and (**B**) shoots of non-heading Chinese cabbage grown hydroponically under control (0 μM) and high Cd treatments T_1_ and T_2_ (10 and 20 μM respectively). Bars show mean values ± SD (*n* = 3). Different letters ‘’a’’ and ‘’b’’ represent statistical significant differences among different varieties under the same treatment according to Tukey’s post-hoc test (*p* < 0.05).

To confirm the Cd accumulation characteristics of the three cultivars in Cd-contaminated soil, the Cd concentration in the hydroponics experiment was set according to the Cd content in the tested soil, and the Cd treatment was performed for 14 days; the experimental results are shown in Figure 2A,B. Cd accumulation in both the roots (Figure 2A) and shoots (Figure 2B) of DF2H, CB, and HLQX cultivars under T_1_ and T_2_ were significantly higher than that in the control, and Cd accumulation in the roots and shoots of the DF2H cultivar was significantly higher than that in the CB and HLQX cultivars (Figure 2A,B). Therefore, we ascertained that DF2H is a high-Cd-accumulation cultivar, whereas CB and HLQX are low-Cd accumulation cultivars. Notably, the results were consistent with the field-culture experiments.

### 3.4. Differences in Cd Uptake in the Three Cultivars under Low Cd Concentration

**Figure 3:**
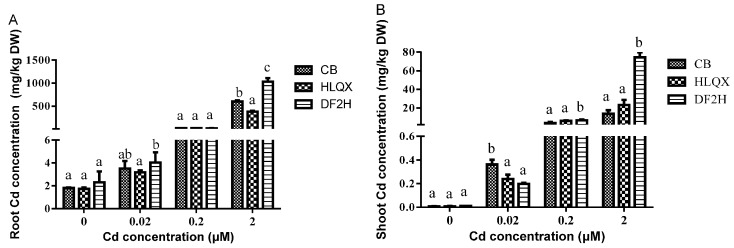
Cd content in the (**A**) roots and (**B**) shoots of non-heading Chinese cabbage grown hydroponically under control (0 μM) and low Cd treatments T_3_, T_4_ and T_5_ (0.02, 0.2 and 2 μM respectively). Bars show mean values ± SD (*n* = 3). Different letters ‘’a’’, ‘’b’’ and ‘’c’’ represent statistical significant differences among different varieties under the same treatment according to Tukey’s post-hoc test (*p* < 0.05).

Soil environment, namely the makeup and management of soil, is correlated with human health. To explore the Cd accumulation of the three cultivars in various soils, we used four treatments in our hydroponics experiments: the control (without Cd), T_3_ (0.02 μM Cd), T_4_ (0.2 μM Cd), and T_5_ (2 μM Cd) according to the MPC of total Cd (0.3 mg/kg, about 2.6 μM) from the EQSSC (GB 15618-2008). The Cd accumulation of the three cultivars increased with an increase in Cd concentration, and the Cd content in DF2H was significantly higher than that in CB and HLQX at a Cd concentration of 2 μM (Figure 3). These results were consistent with the results of field screening and high Cd concentration hydroponics. In addition, the Cd contents in the shoots (Figure 3B) of all three cultivars under the control, T_3_, and T_4_ treatments below the criteria for the Maximum Level of Contaminant in Food (GB2762-2012) of the NSFSC (0.2 mg/kg FW). Under treatment T_5_, the low-Cd-accumulation cultivars CB and HLQX remained below the criteria for the Maximum Level of Contaminant in Food (GB2762-2012) of the NSFSC (0.2 mg/kg FW), whereas in the high Cd-accumulation cultivar DF2H, Cd accumulation was much higher than the Maximum Level of Contaminant in Food (GB2762-2012) of the NSFSC (0.2 mg/kg FW). Therefore, the Cd content in Chinese cabbage grown in these three kinds of soils exceeded the standard and would threaten human health. The Cd content of the two low-Cd-accumulation cultivars was normal and therefore could be consumed normally. Based on these results, the two cabbage cultivars, CB and HLQX, were selected as the CSCs, meeting the MPC allowed under different treatments. It can be seen from Figure 3A that cabbage roots have significant accumulation of Cd, which increases with an increase in Cd concentration.

### 3.5. Mechanism Underlying Cd Accumulation

**Figure 4:**
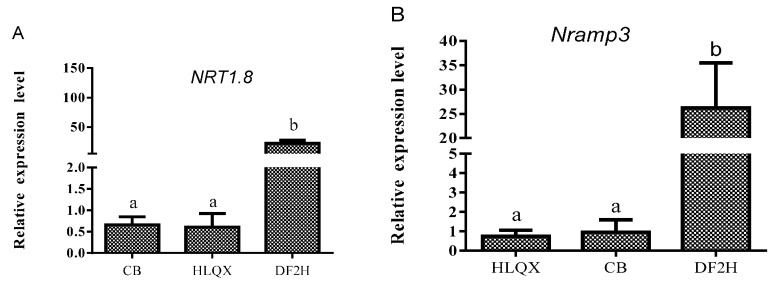
Quantitative RT-PCR analysis of (**A**) *NRT1*.*8* and (**B**) *Nramp3* transporter genes expression in roots of low (HLQX and CB) and high (DF2H) Cd accumulation cultivars of Chinese cabbage under control (Cd, 0 μM) conditions. The y-axis shows the relative expression level normalized to *Actin* (as an internal control). Bars show mean values ± SD (*n* = 3). Different letters ‘’a’’ and ‘’b’’ represent statistical significant differences among different varieties according to Tukey’s post-hoc test (*p* < 0.05).

The above-mentioned experiments identified that Cd accumulated in the shoots and roots of Chinese cabbage grown in the Cd-contaminated environment, but that the Cd contents in the cultivars were different. To explore the reasons for this difference, we first analyzed the Cd TF, which is a parameter that reflects the ability of plants to transfer HMs from their roots to shoots. The differences in TFs among cultivars were insignificant (Table 3), which means that the difference in Cd content among cultivars was due to the characteristics of Cd accumulation rather than translocation restrictions. Therefore, we selected cabbage roots as the material to analyze the expression of genes involved in Cd uptake by qRT-PCR. The expression of *NRT1*.*8* and *Nramp3* was significantly higher in the high-accumulation cultivar DF2H than in the low-accumulation cultivars CB and HLQX (Figure 4). Therefore, the difference in Cd accumulation between the high- and low-accumulation Chinese cabbages may be due to differences in the expression of genes related to Cd uptake.

## 4. Discussion

### 4.1. Screening of High- and Low-Accumulation Cd Varieties of Chinese Cabbage

In recent years, suburb farmland as the main source of urban food has become one of the largest areas affected by HM effluence. The HMs in soil accumulate in food through the food chain and subsequently harm human health. However, the processes for controlling HM contamination in soil are time-consuming and complicated; therefore, the most effective remedy is likely the breeding of low-HM-accumulating cultivars. Many studies have confirmed that the absorption of HMs significantly varies in different cultivars of crops. With regard to cabbage cultivars, Chen et al. [31] found significant differences in Cd uptake in 60 cabbage cultivars under different Cd concentrations via a pot-culture experiment, and screened out cultivars that were low-Cd accumulators. Similar findings to ours have also been demonstrated by Liu et al. [22], who screened 40 Chinese cabbage CSCs in Cd-contaminated soil. 

Due to the uncontrollable conditions in wild field experiments and the factors that greatly affect the growth of vegetables such as temperature, light, and precipitation, our study integrated the data of three screening experiments and identified CB and HLQX as CSCs, and DF2H as the high-Cd-accumulation cultivar. To make certain that the screened cultivars had adapted to the various soils with different Cd contamination levels, high and low Cd concentrations were used in the hydroponics experiments. The results showed that in soils with a low degree of Cd pollution, three cultivars met the requirement for the national standard and were not harmful to human health. In the soils with high levels of Cd pollution, two CSCs (CB and HLQX) met the national standard and were suitable for planting, whereas DF2H exceeded the MPC of the national standard and was not suitable for planting.

This study screened suitable cultivars of Chinese cabbage for planting in different contaminated soils by field-culture and hydroponics experiment, which are of practical significance to the selection of cabbage cultivars in cultivation and the health of residents in the contaminated area. However, a soil’s environment is complex, and metal ions are more available in water than in soil. As such, this study imitated the Cd concentration in soils with different Cd pollution levels, but the screened cultivars needed to be further verified by soil-culture experiments using different Cd concentrations.

### 4.2. Preliminary Study on the Cd Accumulation Mechanism in Chinese cabbage

The control of Cd in vegetables is closely related to human health. Therefore, it is necessary to explore the mechanism of Cd accumulation in vegetables. In this study, we analyzed the differences in translocation factors among different cultivars of Chinese cabbage and the expression differences in Cd-uptake-related genes. The results showed that the difference in Cd content in Chinese cabbage cultivars was not due to a translocation limitation, but rather was caused by the differences in Cd-absorption-related genes (Figure 4). Studies on Cd transporter genes (*Nramp3*, *NRT1.8*, and other related transporters in model plants (rice or Arabidopsis) [17,20,21] have revealed their essential roles for Cd transport with or without Cd treatments and comparatively to Chinese cabbage, it can be anticipated that variable expression of such genes in different cultivars may be associated with low- or high-Cd uptake competences. This study provides a theoretical basis for the further study of the mechanism underling Cd accumulation in Chinese cabbage and the screening of cabbage varieties by molecular methods.

## 5. Conclusions

The low-Cd-accumulation cultivars CB and HLQX were obtained by screening 35 cultivars of Chinese cabbage. The Cd content of the cultivars met the food safety standard, even those grown in poor soils with serious Cd pollution. In addition, it was found that the Cd content of both Cd low- and high-accumulation cultivars was in compliance with food safety standards when planted in soils with low Cd pollution levels. The differences in Cd accumulation in different cultivars were related to the expression of *Nramp3*, and Cd accumulation in the same cultivar increased with the increase in Cd concentration in the growth environment; correspondingly, it can be accentuated that the *NRT1.8* expression can also be correlated to Cd accrual variation among the studied cultivars.

## Figures and Tables

**Table 1:** Chinese cabbage cultivars used in the experiments.

NO.	Name		NO.	Name	
1	Wuxibai	(WXB)	19	Heidatou	(HDT)
2	Nanjinghuoxing	(NJHX)	20	Ribenhuawang	(RBHW)
3	Shanghaiaikangqing	(SHAKQ)	21	Jingguan	(JG)
4	Xiaomei	(XM)	22	Jinguan	(JG1)
5	Xiawang	(XW)	23	Siyuebai	(SYB)
6	Liuyueman	(LYM)	24	Dongfang2hao	(DF2H)
7	Hualvqingxiu	(HLQX)	25	Xiaqiang	(XQ)
8	Aijaohuang	(AJH)	26	Canbai	(CB)
9	Lvyouxing	(LYX)	27	Huangguan	(HG)
10	Qibaoqing	(QBQ)	28	Sijiqing	(SJQ)
11	Erzhuangbai	(EZB)	29	Sijiquanneng	(SJQN)
12	Changgengbai	(CGB)	30	Lvqingyihao	(LQYH)
13	Jimaocai	(JMC)	31	Siyueman	(SYM)
14	Kangre 605	(KR 605)	32	Sijixiaobaicai	(SJXBC)
15	Shanghaiqing	(SHQ)	33	Zhongqibai	(ZQB)
16	Xiaoairen	(XAR)	34	Gaoganbai	(GGB)
17	Wuyueman	(WYM)	35	Aijiaoqing	(AJQ)
18	Suzhouqing	(SZQ)			

**Table 2:** Physical-chemical properties of tested topsoil.

Soil Property	Field-Culture Soil
pH	6.13
Total Cd (mg/kg DW)	2.42
Total Pb (mg/kg DW)	449.19
Available Cd (mg/kg DW)	1.73 ± 0.12
Available Pb (mg/kg DW)	70.39 ± 11.96
Organic matter (mg/kg DW)	5.12
Ammonium (mg/kg DW)	25.67
Available P (mg/kg DW)	37.54
Available K (mg/kg DW)	49.89

**Table 3:** Translocation factors (TFs) of different concentrations of Cd in different cultivars of non-heading Chinese cabbage.

TFs		Cultivars		
		CB	HLQX	DF2H
Low-Cd treatments	0.02 μM	1.103	0.075	0.049
	0.2 μM	0.191	0.28	0.291
	2 μM	0.035	0.038	0.071
High-Cd treatments	10 μM	0.10	0.07	0.06
	20 μM	0.15	0.12	0.12
